# Lipoxin A_4_ Prevents the Progression of De Novo and Established Endometriosis in a Mouse Model by Attenuating Prostaglandin E_2_ Production and Estrogen Signaling

**DOI:** 10.1371/journal.pone.0089742

**Published:** 2014-02-24

**Authors:** Rajesh Kumar, Anne-Catherine Clerc, Ilaria Gori, Ronan Russell, Chiara Pellegrini, Lerisa Govender, Jean-Christophe Wyss, Dela Golshayan, Geraldine O. Canny

**Affiliations:** 1 Mucosal Immunity Laboratory, Department of Gynecology, Obstetrics and Medical Genetics, Lausanne University Hospital and University of Lausanne, Lausanne, Switzerland; 2 Transplantation Centre and Transplantation Immunopathology Laboratory, Department of Medicine, Lausanne University Hospital and University of Lausanne, Lausanne, Switzerland; National Cancer Center, Japan

## Abstract

Endometriosis, a leading cause of pelvic pain and infertility, is characterized by ectopic growth of endometrial-like tissue and affects approximately 176 million women worldwide. The pathophysiology involves inflammatory and angiogenic mediators as well as estrogen-mediated signaling and novel, improved therapeutics targeting these pathways are necessary. The aim of this study was to investigate mechanisms leading to the establishment and progression of endometriosis as well as the effect of local treatment with Lipoxin A_4_ (LXA_4_), an anti-inflammatory and pro-resolving lipid mediator that we have recently characterized as an estrogen receptor agonist. LXA_4_ treatment significantly reduced endometriotic lesion size and downregulated the pro-inflammatory cytokines IL-1β and IL-6, as well as the angiogenic factor VEGF. LXA_4_ also inhibited COX-2 expression in both endometriotic lesions and peritoneal fluid cells, resulting in attenuated peritoneal fluid Prostaglandin E_2_ (PGE_2_) levels_._ Besides its anti-inflammatory effects, LXA_4_ differentially regulated the expression and activity of the matrix remodeling enzyme matrix metalloproteinase (MMP)-9 as well as modulating transforming growth factor (TGF)-β isoform expression within endometriotic lesions and in peritoneal fluid cells. We also report for first time that LXA_4_ attenuated aromatase expression, estrogen signaling and estrogen-regulated genes implicated in cellular proliferation in a mouse model of disease. These effects were observed both when LXA_4_ was administered prior to disease induction and during established disease. Collectively, our findings highlight potential targets for the treatment of endometriosis and suggest a pleotropic effect of LXA_4_ on disease progression, by attenuating pro-inflammatory and angiogenic mediators, matrix remodeling enzymes, estrogen metabolism and signaling, as well as downstream proliferative pathways.

## Introduction

Endometriosis is a gynecological disorder, defined as the presence of endometrial-like tissue outside the uterine cavity, primarily on the pelvic peritoneum, ovaries, pouch of Douglas and rectovaginal septum. Endometriosis affects approximately 10% of women of reproductive age and is associated with local inflammation, the main clinical features being chronic pelvic pain, pain during intercourse and infertility [1]. The pathophysiology of this estrogen-dependent disease is enigmatic, with a poor correlation between the observed severity of the disease and the reported symptoms [2], [3]. The medical treatment of endometriosis includes progestagens, gonadotropin-releasing hormone agonists and oral contraceptives. However, the use of these therapeutics is limited due to the high cost, adverse side effects and recurrence of the disease after discontinuation of therapy [4], [5]. Thus, there is an unmet need for new drugs which inhibit the progression of endometriosis and alleviate pain and infertility, without affecting ovulation.

The progression of endometriosis is characterized by marked alternations in estrogen metabolism and pro-inflammatory mediator levels and activity [6], [7]. In the peritoneal cavity activated macrophages are the major source of IL-1β, IL-6, IL-8 and TNF-α and altered expression of these proinflammatory cytokines has been described in the peritoneal fluid of women with endometriosis. The disease is not only characterized by the presence of inflammatory lesions, but also by abnormal proliferation and growth of ectopic endometrial tissue [8], [9]. In endometrial implants, vascular endothelial growth factor (VEGF) and transforming growth factor-beta (TGF-β) induce neo-vascularization [2], [6], [10]. Moreover, IL-1β and TNF-α have been shown to increase the expression of MMPs in endometriotic implants, promoting matrix remodeling [11].

Lipoxins are endogenous eicosanoids, generally produced via a transcellular biosynthetic pathway, which exhibit both anti-inflammatory and pro-resolving properties [12]. LXA_4_ mediates anti-inflammatory activities through multiple receptors *in vivo*, the best characterized being lipoxin A_4_ receptor (ALX)/formyl peptide receptor 2 (FPR2) [13]. LXA_4_ binds with high affinity to ALX/FPR2, expressed by myeloid and non-myeloid cells [14], [15]. Other more recently characterized receptors are GPR32 [16] and the arylhydrocarbon receptor [17], the later study having used murine hepatoma cells. We have recently demonstrated that this lipid mediator is estrogenic *in vitro* and *in vivo*, binding to and signaling via ERα in endometrial epithelial cells [18], where it can counteract some 17β-estradiol (E2)-mediated responses. Lipoxins are known to be potent chemo-attractants for peripheral blood monocytes and induce non-phlogistic phagocytosis of apoptotic neutrophils by macrophages [19]. Macrophage recruitment would thus lead to the resolution of inflammation which is important for the restoration of tissue homeostasis [20]. However, macrophages, being a major source of prostaglandin E_2_ (PGE_2_)_,_ may also promote endometriosis [21]. Besides being implicated in symptomatic inflammation and pain, PGE_2_ plays a key role in the pathology by inducing E2 production in endometriotic cells.

Recent data have suggested a protective role of LXA_4_ in the development and progression of experimental endometriosis in mice [22], [23]. However, the mechanisms by which LXA_4_ mediates this action remain poorly understood. The aim of the present study was to better understand the mechanisms underlying this effect and to establish whether LXA_4_ is protective for established endometriosis, focusing on inflammatory and angiogenic mediators as well as genes involved in estrogen production and in downstream signaling.

## Materials and Methods

### Animals

All experimental procedures were approved by Canton de Vaud veterinary authorizations (protocol numbers 1884.2 and 2655.0). All surgery was performed under anesthesia and all efforts made to minimize suffering. Seven week old wild-type female C57BL/6J mice were purchased from Elevage Janvier, France, randomly housed ten per cage for one week prior to surgery and fed on mouse diet and water *ad libitum*. The animal house temperature was maintained at 26°C with light/dark cycles of 12 h under controlled humidified conditions. Age-matched mice were synchronized prior to all experiments, by keeping them in mixed cages for at least 1 week prior to surgery (Lee-Boot Effect). Mice were then caged 5 per cage, randomly assigned to each experimental group and underwent surgery at 8 weeks of age. Cycle stage was checked by microscopic examination of vaginal swabs at day 2, day 10 and day 21 and at day 21 the majority of mice were in estrus stage.

### Surgical induction of endometriosis and *in vivo* treatment

The surgical method to induce endometriosis was modified from Vernon and Wilson [24] and Cumming and Metcalf [25]. Mice were randomly divided into three groups; sham, vehicle-treated with endometriosis (‘Veh’) and LXA_4_-treated with endometriosis (‘LXA_4_’). Briefly, mice were anesthetized using Xylazine (10 mg/kg, Rompun, Bayer Healthcare, Germany) and Ketamine (100 mg/kg, Ketavet, Phamacia&Upjohn GmbH, Germany) and their weight was recorded. Uterine tissue from one donor mouse was transplanted into two recipient mice. Mice underwent laparotomy by a midventral incision. Uterine horns were excised from age-matched syngeneic donor mice and kept in 37°C pre-warmed DMEM-F12 (Invitrogen, Switzerland) medium supplemented with penicillin (100 U/mL, Sigma) and streptomycin (100 µg/mL, Sigma) in petri dishes, opened longitudinally and six uniform, full thickness 2-mm punches were made using a dermal biopsy punch (Stiefel, Germany). Six endometrial biopsies were sutured to the peritoneal wall of recipient mice, three on each side. Sham mice were operated on identically except that silk sutures alone were placed on the peritoneal wall in the same positions as other groups. The abdominal incision was closed by a continuous suture and the skin with individual knots. Mice were kept warm and maintained under observation until full recovery.

LXA_4_ (Calbiochem, USA) was administered once daily by intraperitoneal (IP) injection at 5 µg/kg, a concentration demonstrated to be effective in preliminary experiments, in 200 µl sterile phosphate buffered saline (PBS, Laboratrium Dr. Bichsel AG, Switzerland). Sham and Veh groups received 200 µl EtOH/PBS IP in an identical manner. To assess the effect of LXA_4_ on *de novo* endometriosis, 5 µg/kg was administered once daily by IP injection 1 day prior (D−1) to surgery with treatment given daily thereafter until day 20. To assess the effect of LXA_4_ on established endometriosis treatment was commenced 6 days after disease induction (D+6) and performed in an identical manner. Mice were monitored daily and no adverse effects were observed based on food consumption, body weight, grooming behavior, or physical activity.

### Evaluation of endometriotic lesions and sample collection

Mice were weighed and sacrificed by cervical dislocation under anaesthesia on the morning of day 21. 1 ml of sterile PBS was injected IP, the abdominal cavity was gently massaged and the peritoneal fluid was collected by centrifugation. Peritoneal lavage supernatants were transferred to sterile eppendorfs and stored at −80°C for ELISAs, while cell pellets were stored at −80°C for subsequent qRT-PCR analysis. Ectopic endometrial lesions were measured using calipers in two perpendicular diameters and the volume was calculated (V = 4/3πr^2^R, where r and R are two perpendicular radii and r<R). Lesions were flash frozen in liquid Nitrogen for qRT-PCR and stored at −80°C or fixed in 10% neutral buffered formalin for 24 h and embedded in paraffin for immunohistochemistry.

### RNA isolation and qRT-PCR

Total RNA from individual endometriotic lesions and peritoneal fluid cells (PFCs) were extracted with TRIzol reagent (Invitrogen, USA) as per the manufacturer's instructions. 5–10 mg endometriotic lesion tissue and pelleted PFCs were homogenized using a polytron (VWR International AG, Switzerland) in 600 and 800 µl of TRIzol respectively, followed by chloroform/isopropanol extraction. RNA concentration and purity were checked using a Nanodrop ND2000 spectrophotometer (Thermo Scientific, USA). RNA samples with 260/280 nm absorbance ratio of ∼2 (∼1.8 for cDNA) were subjected to expression analyses. DNase (Promega, USA) treatment was followed by reverse transcription (RT), performed using the iScriptTM cDNA Synthesis kit (Bio-rad Laboratories, USA) according to the manufacturer's instructions. Quantitative PCR was performed using a Rotor-Gene 6000 (Corbett Research, Australia) instrument and SYBR Green technology (SensiMix^TM^ SYBR kit, Quantace). Intron spanning and exon-specific primers were designed manually and synthesized by Microsynth (Switzerland). Primers were initially validated by executing standard curves (primer with single melting curve peak and amplification efficiency >80% were considered) and by confirming product size on an agarose gel with ethidium bromide staining. Primer sequences are listed in Table 1. The ΔΔCt method [26] was used to calculate relative changes in mRNA expression. Data were normalized with GAPDH as housekeeping gene and 100% assigned to the vehicle-treated experimental group.

**Table 1:** 

Gene Symbol	Forward Sequence (5′–3′)	Reverse Sequence (5′–3′)
m CCND-1	CACCAATCTCCTCAACGACC	CTCACAGACCTCCAGCATCC
m COX-2	CAGGTCATTGGTGGAGAGGTG	TGCTCATCACCCCACTCAGG
m CYP19a1	CATTATCAGCAAGTCCTCAAGC	GAGGGTCAACACATCCACG
m ERα	GATGAAAGGCGGCATACGG	CAGGGCTATTCTTCTTAGTGTGC
m FPR2	GTGTGCTGGGCAATGGAC	CTGAGCCCAGACTGGATGC
m GAPDH	GTCGGTGTGAACGGATTTG	AAGATGGTGATGGGCTTCC
m GREB-1	GCACCACTTTGTTTTCAGCC	GTTGTCGGTGTGAAGGTTGG
m HIF-1α	TCACCAGACAGAGCAGGAAA	GTCACCTGGTTGCTGCAATA
m IL-1β	GGGCCTCAAAGGAAAGAATC	CTCTGCTTGTGAGGTGCTGA
m IL-6	AGAAGGGCCTGGAATGAAAC	AAGACCCTGCTGGAACAAGA
m MMP-2	CCAAGGTGGAAATCAGAGACA	CGAAGGGC ATACAAAAGCAAC
m MMP-3	CCTGGAACAGTCTTGGCTC	CCTTGGCTGAGTGGTAGAGTC
m MMP-9	GCCCTGGAACTCACACGACA	TTGGAAACTCACACGCCAGAAG
m MRP4	CCTGTTCCAACTGTGTATCTGTC	GCACCATCTCACCATCTTTG
m c-Myc	GCAGACAGCCACGACGATG	CTCGCTCTGCTGTTGCTGG
m TGF-β1	GAACCAAGGAGACGGAATACAG	CCATGAGGAGCAGGAAGG
m TGF-β2	CCTGCTAATGTTGTTGCCC	CAATGTAAAGAGGGCGAAGG
m TGF-β3	CGCAACCTGGAGGAGAACTG	CGTGCTATGGGTTGTGTCTG
m VEGF	AGTCCCATGAAGTGATCAAGTTCA	ATCCGCATGATCTGCATGG

### Cytokine and PGE_2_ quantification

Peritoneal lavage supernatants were thawed on ice and centrifuged at 12000rpm for 5′ at 4°C. The Biorad protein quantification kit (Bio-Rad Laboratories, USA) was used to normalize protein content. Samples containing 50 µg of total protein were assayed. VEGF and IL-1β cytokine levels were measured by ELISA according to the manufacturer's instructions (R&D Systems, USA).

Peritoneal fluid samples were diluted using water and methanol. The mixture was acidified with 2N HCl to pH 3.5 and applied to a C_18_ cartridge (Sep-Pak Classic, Waters®, IT) which was preactivated with methanol and water. The columns were washed with water and hexane, eluted by adding methyl-formate (1∶1 methanol and formic acid) and evaporated under a nitrogen stream. The residue was reconstituted in EIA Buffer and PGE_2_ quantification was performed using a monoclonal EIA kit (Cayman Chemical company, USA). Briefly, standards and samples containing 250 μg total protein were incubated overnight at 4°C in a plate pre-coated with goat anti-mouse IgG, with PGE_2_ Tracer (PGE_2_-Acetylcholineesterase conjugate) and mouse anti-PGE_2_ monoclonal antibody. The plate was subsequently washed, Ellman's reagent (5,5′-dithio-bis-(2-nitrobenzoic acid) and acetylcholine added and the absorbance was read at 405 nm.

### Histology and immunohistochemistry

Formalin fixed and paraffin embedded tissues were cut into 5 µm sections and mounted on SuperFrost Ultras Plus glass slides (Thermo Scientific, Germany). For gross examination sections were stained with hematoxylin (Merck Chemicals, Switzerland) and eosin (HE staining) following standard protocols and mounted using permanent mounting medium (Eukitt, Sigma-Aldrich, Switzerland). For immunohistochemical staining, sections were deparaffinized (55°C for 20′) and rehydrated by serial bathing in xylene and EtOH solutions, washed in PBS for 5′ and antigen retrieval performed using Tris-EDTA pH 9 solution. Slides were incubated in 3% H_2_O_2_ solution for 10′ to block endogenous peroxidases, washed, incubated at RT in blocking buffer (PBS-4% BSA-0.05% Tween 20) for 30′ followed by washing. Diluted primary antibody (in PBS-1% BSA) was applied to the slides for 90′. The following antibodies were used: rabbit anti-mouse COX-2 (Cayman Chemical company, USA) and anti-mouse ERα (Serotec, USA). For COX-2 immunohistochemistry, inflamed joint sections from mice with induced arthritis were used to optimize the staining and for ERα, eutopic uterus sections were used as a positive control. Negative control sections were incubated alongside with the respective isotype control antibodies in dilution buffer, to control for specificity. After washing, horseradish peroxidase-conjugated secondary antibodies (Cell Signaling Technology, USA) was added for 20′ with the diaminobenzidine (DAB), Dako kit, Denmark) substrate. Counterstaining was performed with hematoxylin, followed by dehydration steps and mounting. Slides were analyzed with a Nikon Eclipse E800 microscope and digital DXM1200 camera using ACT-1 software.

### Zymography

Gelatinolytic activities of MMP-2 and MMP-9 were assessed using zymography. Briefly, 20 µl of peritoneal fluid sample, whose protein content had been normalized as detailed above, was mixed with non reducing loading buffer, loaded and electrophoresed through 8% SDS polyacrylamide gels containing 0.1% gelatin. Gels were then washed with renaturation buffer (2.5% Triton X-100) for 40′ followed by incubation in zymogram incubation buffer (50 mM Tris pH 7.8, 0.15 M NaCl, 10 mM CaCl_2_, 0.02%) for 20 h at 37°C. Subsequently, staining with 0.25% Coomassie G250, 30% methanol, 10% acetic acid for 30′ and destaining with 30% methanol, 10% acetic acid was performed until white bands appeared over blue background. Gels were scanned and white bands were quantified using Gel Analyzer software. The relative enzymatic activity was calculated as a percentage, samples from Veh-treated mice being assigned 100%.

### Data analysis & statistics

At least 3 independent experiments were performed for each set of data with 10–15 mice per experimental group depending on the requirements of the experiments. When repeated experiments yielded similar results, the results of one representative experiment are shown. Results are expressed as mean ± SEM. Statistical analysis was performed using unpaired t-tests for comparisons between 2 groups (analysis of endometriotic lesions, comparing Veh and LXA_4_) and one way analysis of variance (ANOVA) along with Tukey's multiple comparison tests to check the level of significance among multiple treatment groups (analysis of PFCs and PF, comparing sham, Veh and LXA_4_). Graph pad Prism version 6 (San Diego, CA, USA) was used; p<0.05 was considered statistically significant.

## Results

### Treatment with LXA_4_ reduces the growth of surgically induced endometriotic lesions

**Figure 1:**
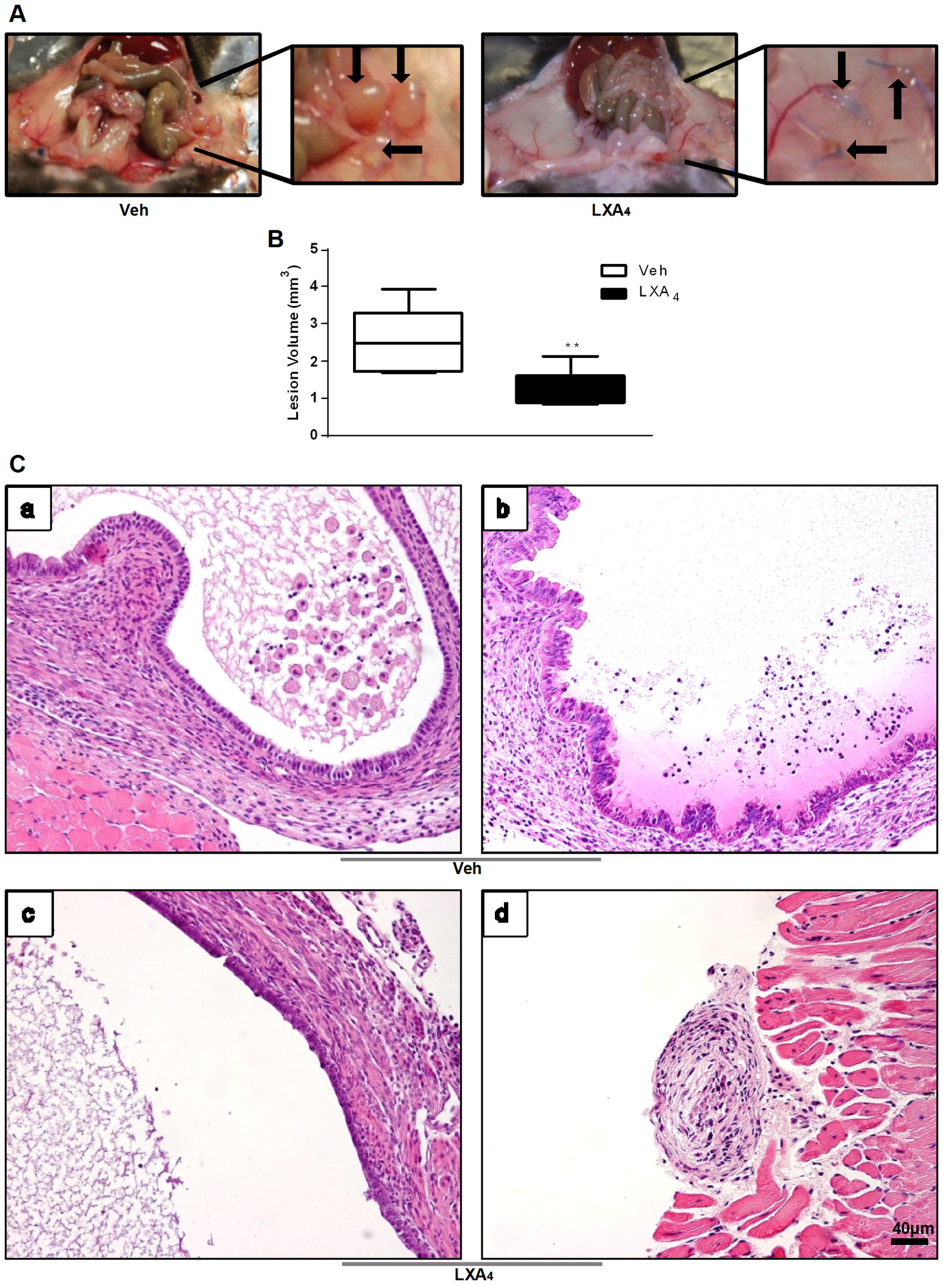
Treatment with LXA_4_ decreases endometriotic lesion growth. **A.** Representative pictures of a mouse with endometriotic lesions treated with vehicle (Veh, left panel) or LXA_4_ (right panel) for 21 days. Six uterine biopsies were implanted on the peritoneal wall, 3 on each side, in a minimum of 20 mice which were randomly divided into two groups: Veh- and LXA_4_-treated. Implanted tissues grew into enlarged cyst-like lesions by 21 days after endometriosis induction in the Veh group, while LXA_4_ treatment significantly reduced lesion size. **B**. Graphical representation of endometriotic lesions volume, in mm^3^ ± SEM (**p<0.01). **C.** Morphology of formalin-fixed, paraffin-embedded lesion sections stained with HE. Upper panels a. and b. Vehicle-treated mouse with endometriosis. Lower panels c. and d. LXA_4_-treated mouse with endometriosis. Images are representative of ten biological replicates.

Wild-type female C57BL/6J mice underwent surgery at 8 weeks of age and developed endometriotic lesions within a few days, which increased in size over time. We analyzed lesion size and gene expression profiles at days 3 and 21 after surgery. As most consistent results were observed at day 21, data from this time point are shown. Three weeks after surgery we examined the presence and growth of endometriotic lesions in the peritoneal cavity of sham-operated control mice, vehicle-treated mice with endometriosis (‘Veh’) and LXA_4_-treated mice with endometriosis (‘LXA_4_’). Veh mice developed cyst-like lesions implanted on the peritoneal wall surrounded by adhesions to adjacent organs, validating our *in vivo* experimental model. No such structures were observed in sham-operated mice. A significant reduction in lesion volume was observed in LXA_4_ compared to Veh mice (Figure 1). Macroscopic histological examination of the lesions from Veh mice confirmed the presence of endometriotic glands surrounded by stroma. Treatment with LXA_4_ resulted in a more rudimentary architecture with less developed glands surrounded by little stroma (Figure 1).

### LXA_4_ attenuates pro-inflammatory and pro-angiogenic mediators in endometriotic lesions and PFCs

**Figure 2:**
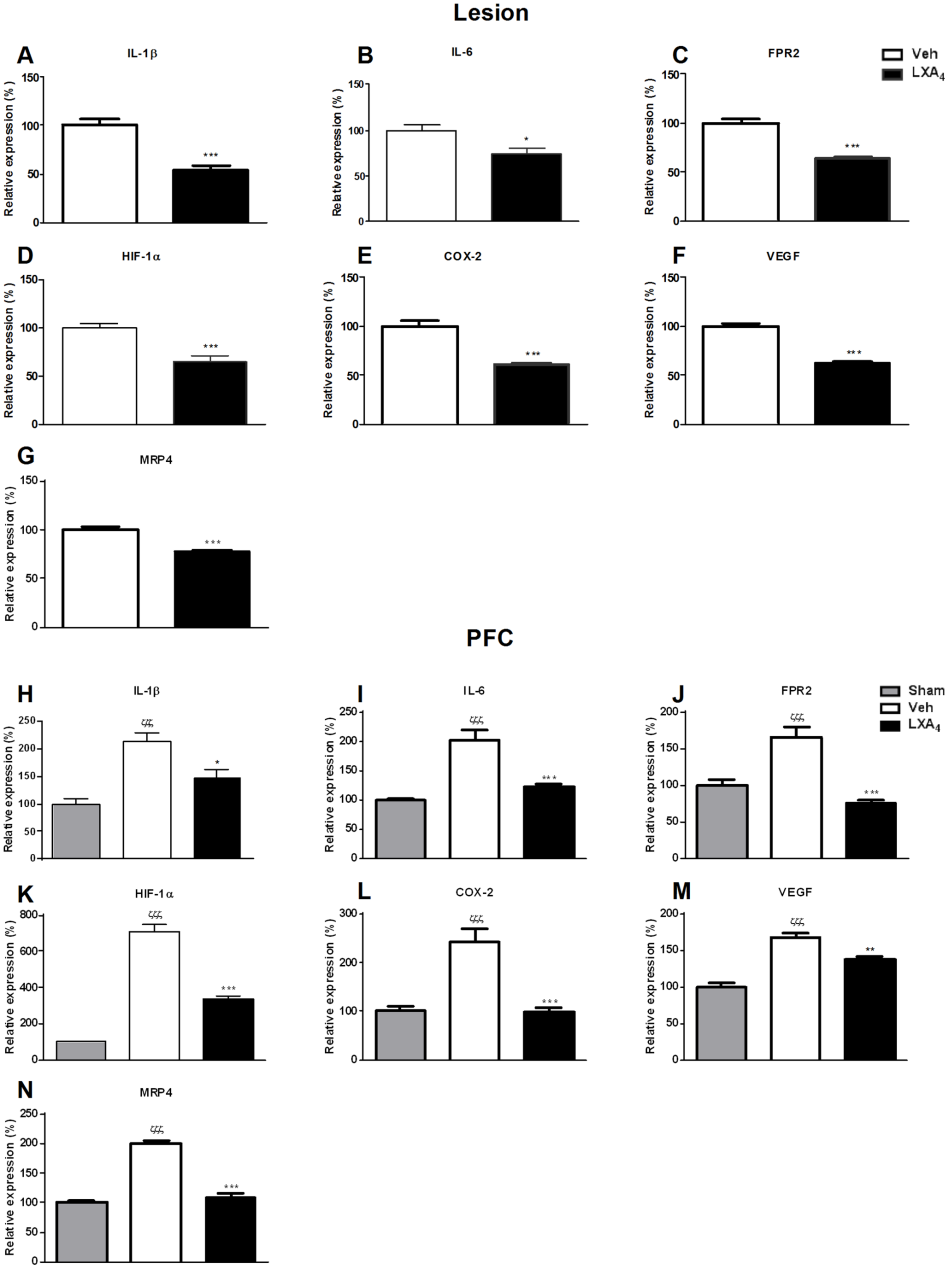
LXA_4_ significantly attenuates the expression of pro-inflammatory cytokines, angiogenic mediators, COX-2 and the PGE_2_ transporter MRP4. **A.** IL-1β, **B.** IL-6, **C.** FPR2, **D.** HIF-1α, **E.** COX-2, **F.** VEGF and **G.** MRP4 mRNA expression in endometriotic lesions was analyzed by qPCR. **H.** IL-1β, **I.** IL-6, **J.** FPR2, **K.** HIF-1α, **L.** COX-2, **M.** VEGF and **N.** MRP4 mRNA expression was similarly quantified in PFCs. Ten mice were used per group (Sham, Veh-, LXA_4_-treatment) and samples analyzed for each individual mouse. Mice were sacrificed 21 days after endometriosis induction and Veh- or LXA_4_-treatment. Endometriotic lesions and PFCs were collected for RNA isolation and qPCR. Data were normalized to GAPDH and are presented as mean ± SEM (*p<0.05, **p<0.01, ***p<0.001 compared to Veh; ζζζ p<0.001 compared to Sham).

We next investigated the mechanisms by which LXA_4_ treatment controlled the development of endometriotic lesions and the resulting inflammatory response. Genes involved in the initiation of inflammatory pathways were analysed by qPCR. At day 21, we observed significantly reduced IL-1β and IL-6 mRNA expression in endometriotic lesions and PFCs from LXA_4_-treated (Figure 2A, 2B, 2H, 2I) compared to Veh mice. In the absence of endometriosis i.e in sham mice, we didn't observe any noticeable effect of LXA_4_ on the main inflammatory mediators in initial experiments (data not shown), and therefore to conserve mice, this group was not included in subsequent experiments. LXA_4_ signals via ALX/FPR2, a G protein-coupled receptor, in many cell types including epithelial cells as well as innate immune cells such as macrophages and neutrophils. This receptor was significantly attenuated by LXA_4_ treatment in both lesions and peritoneal fluid cells (Figure 2C, 2J).

Under hypoxic and inflammatory conditions the Hypoxia-inducible factor-1α (HIF-1α) complex binds to hypoxia responsive elements leading to the transcriptional activation of many target genes including the pro-angiogenic mediator VEGF [27], [28]. Upon LXA_4_ treatment, we observed inhibition of HIF-1α expression (Figure 2D, 2K) and its target gene VEGF (Figure 2F, 2M) in both endometriotic lesions and PFCs, respectively. The activation of VEGF is also regulated by E2 and in turn induces cyclooxygenase-2 (COX-2) expression. LXA_4_ treatment resulted in a significant reduction in COX-2 expression in endometriotic lesions (Figure 2E) and PFCs (Figure 2L). Interestingly, LXA_4_ also downregulated the expression of the multidrug resistance protein 4 (MRP4/ABCC4), which transports PGE_2_ into the extracellular space [29], in endometriotic lesions (Figure 2G) and PFCs (Figure 2N).

We observed consistently elevated transcript levels of pro-inflammatory cytokines (IL-1β, IL-6) as well as ALX/FPR2, HIF-1α, COX-2, VEGF and MRP4 in PFCs of Veh- mice to compared to sham-operated mice (Figure 2H-2N), further confirming the implication of these mediators in the pathogenesis of disease.

### LXA_4_ reduces peritoneal fluid pro-inflammatory cytokine and PGE_2_ levels, as well as COX-2 expression in lesions and peritoneal fluid cells

**Figure 3:**
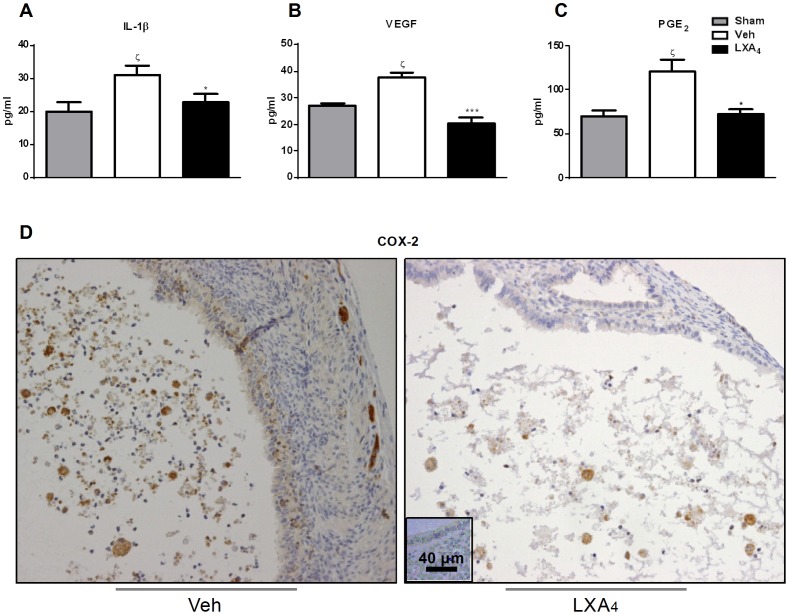
LXA_4_ reduces peritoneal fluid cytokine and PGE_2_ levels as well as the expression of COX-2 in endometriotic lesions. Peritoneal fluid from mice was collected at sacrifice (Sham, Veh-, LXA_4_; 3–5 mice/group) and ELISAs performed to quantify **A.** IL-1β, **B.** VEGF and **C.** PGE_2_ levels. Results are expressed as pg mediator/ml for 50 μg of total peritoneal fluid protein and presented as mean ± SEM (*p<0.05, ***p<0.001 compared to Veh; ζ<0.05 compared to Sham). **D.** Immunohistochemical staining for COX-2 was performed on transverse paraffin-embedded sections of lesions from Veh- and LXA_4_-treated mice. Representative stainings are shown with the negative control in the inset.

To confirm the qPCR data and further investigate the role of inflammation and angiogenesis in the development of endometriosis in our experimental model, IL-1β and VEGF contents in the peritoneal fluid were measured at the protein level by ELISA and found to be higher in veh-treated mice compared to sham (Figure 3A, 3B). Indeed, LXA_4_ treatment resulted in a significant reduction in the levels of these cytokines (Figure 3A, 3B). Moreover, PGE_2_ was significantly higher in Veh-treated mice compared to sham and reduced in the peritoneal fluid of LXA_4_ compared to Veh-treated mice (Figure 3C). We therefore performed immunohistochemical staining for COX-2 on endometriotric lesions. Intense COX-2 staining was mainly observed in the cytoplasm of stromal cells and macrophages, the latter cell type was also confirmed as F4/80 positive by immunohistochemical analysis (data not shown). LXA_4_ treatment attenuated the expression of COX-2 protein in endometriotic lesions in comparison to Veh-treated mice (Figure 3D).

### LXA_4_ modulates the expression of matrix remodelling mediators in endometriotic lesions and PFCs

**Figure 4:**
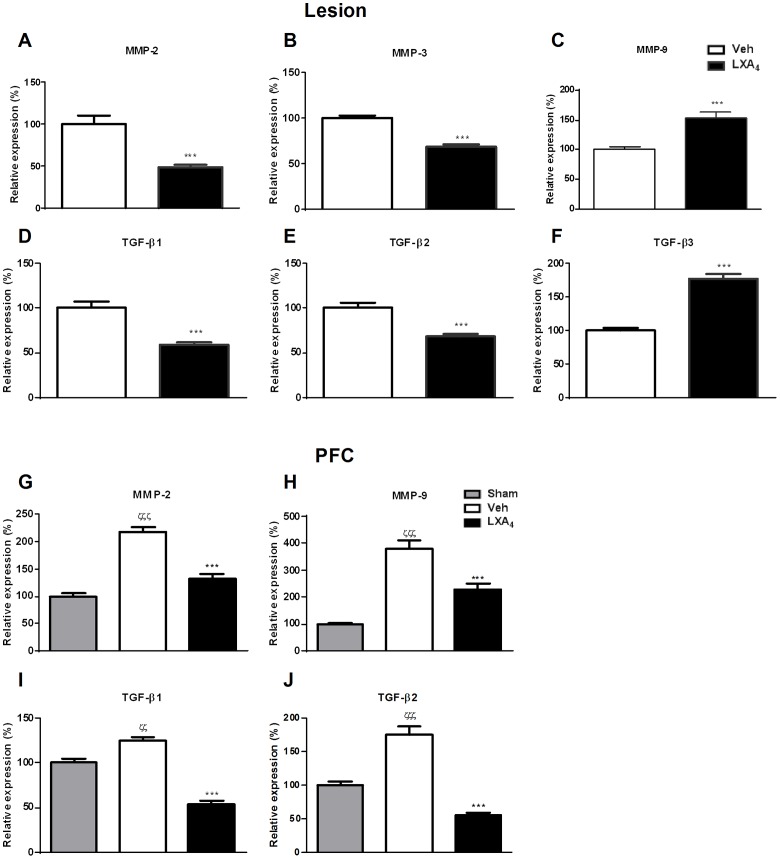
LXA_4_ significantly inhibits the expression of matrix remodeling genes. **A.** MMP-2, **B.** MMP-3, **C.** MMP-9, **D.** TGF-β1, **E.** TGF-β2, **F.** TGF-β3, **G.** MMP-2, **H.** MMP-9, **I.** TGF-β1, **J.** TGF-β2 gene expression in lesions and PFCs. Ten mice were used per group (Sham, Veh-, LXA_4_-treatment) and sacrificed 21 days after endometriosis induction. Lesions and PFCs were collected for qPCR analyses. Data were normalized to GAPDH and presented as mean ± SEM (***p<0.001 compared to Veh; ζζ p<0.01, ζζζ p<0.001 compared to Sham).

**Figure 5:**
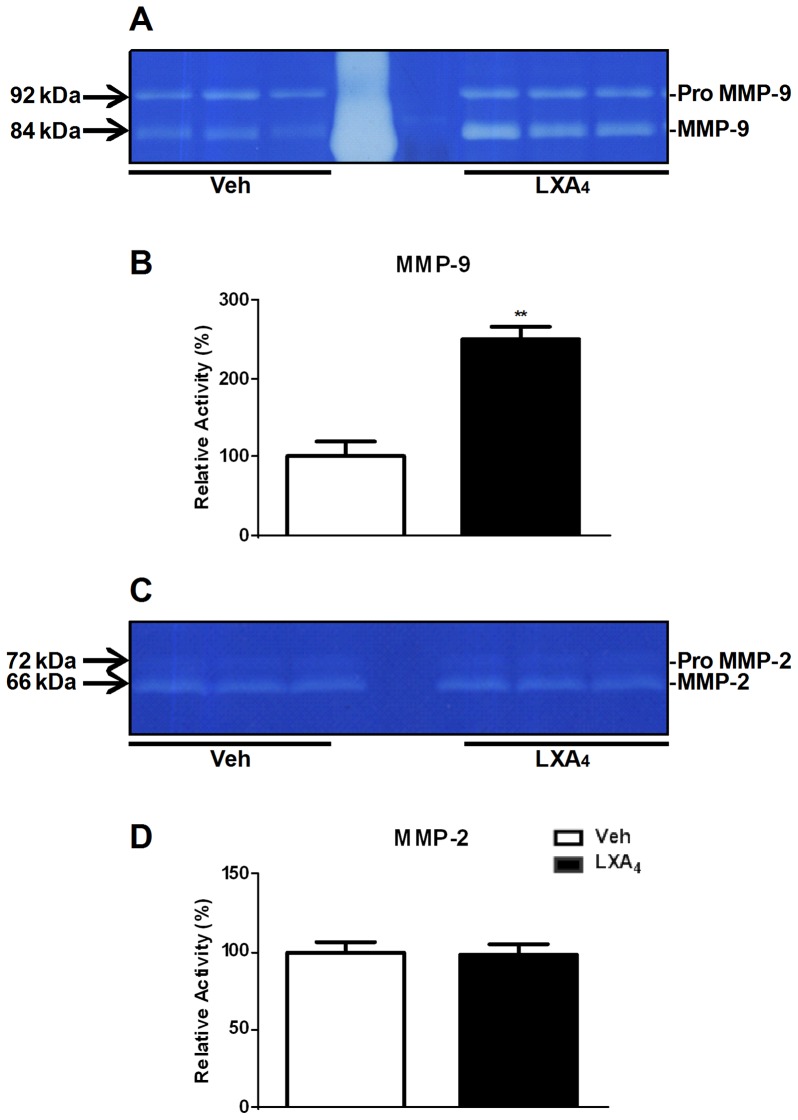
Gelatin zymography analysis of MMP-2 and MMP-9 activity. A. Representative gel showing gelatinolytic activities of **A.** MMP-9, and **C.** MMP-2 in peritoneal fluid of Veh- and LXA_4_-treated mice (3–4 mice/group). Bar graphs represent the densitometric quantification of **B.** MMP-9 and **D.** MMP-2 activity, respectively. Unpaired *t*-tests were performed for comparison with the corresponding Veh-treatment group (**p<0.01).

Matrix metalloproteinases (MMPs) are involved in extracellular matrix degradation. In physiological conditions, macrophages are recruited to the peritoneal cavity to phagocytose the retrograded menstrual tissue [30], [31]. MMPs, and in particular MMP-9, facilitate the degradation of type IV collagen-containing basement membrane of extracellular matrix that separates the epithelial and stromal compartment. In our *in vivo* model, we analyzed MMP expression at day 21 in endometriotic lesions and PFCs. Treatment with LXA_4_ downregulated MMP-2 (Figure 4A) and MMP-3 (Figure 4B) but upregulated MMP-9 (Figure 4C) mRNA expression in endometriotic lesions. The expression of MMP-2 (Figure 4G) and MMP-9 (Figure 4H) was, however, similarly downregulated in PFCs by LXA_4_ treatment. Because of this differential expression, we further analysed MMP-2 and MMP-9 at the functional level. Enzymatic assay of peritoneal fluid samples confirmed increased MMP-9 activity (Figure 5A, 5B) under these conditions, whereas MMP-2 activity remained unchanged (Figure 5C, 5D).

Transforming growth factor beta (TGF-β) is a pleiotropic factor which is implicated in cell proliferation, differentiation, apoptosis, as well as in immune regulation. TGF-β1 and 3 are expressed in epithelial and stromal cells, while TGF-β2 is mainly expressed in the stroma [32]. In endometriotic lesions and PFCs, LXA_4_ treatment reduced TGF-β1 (Figure 4D, 4I) and TGF-β2 (Figure 4E, 4J) expression. However, TGF-β3 (Figure 4F) expression was augmented upon LXA_4_ in endometriotic lesions only, while it was not detected in PFCs.

### LXA_4_ inhibits estrogen regulated genes in endometriotic lesions and PFCs

**Figure 6:**
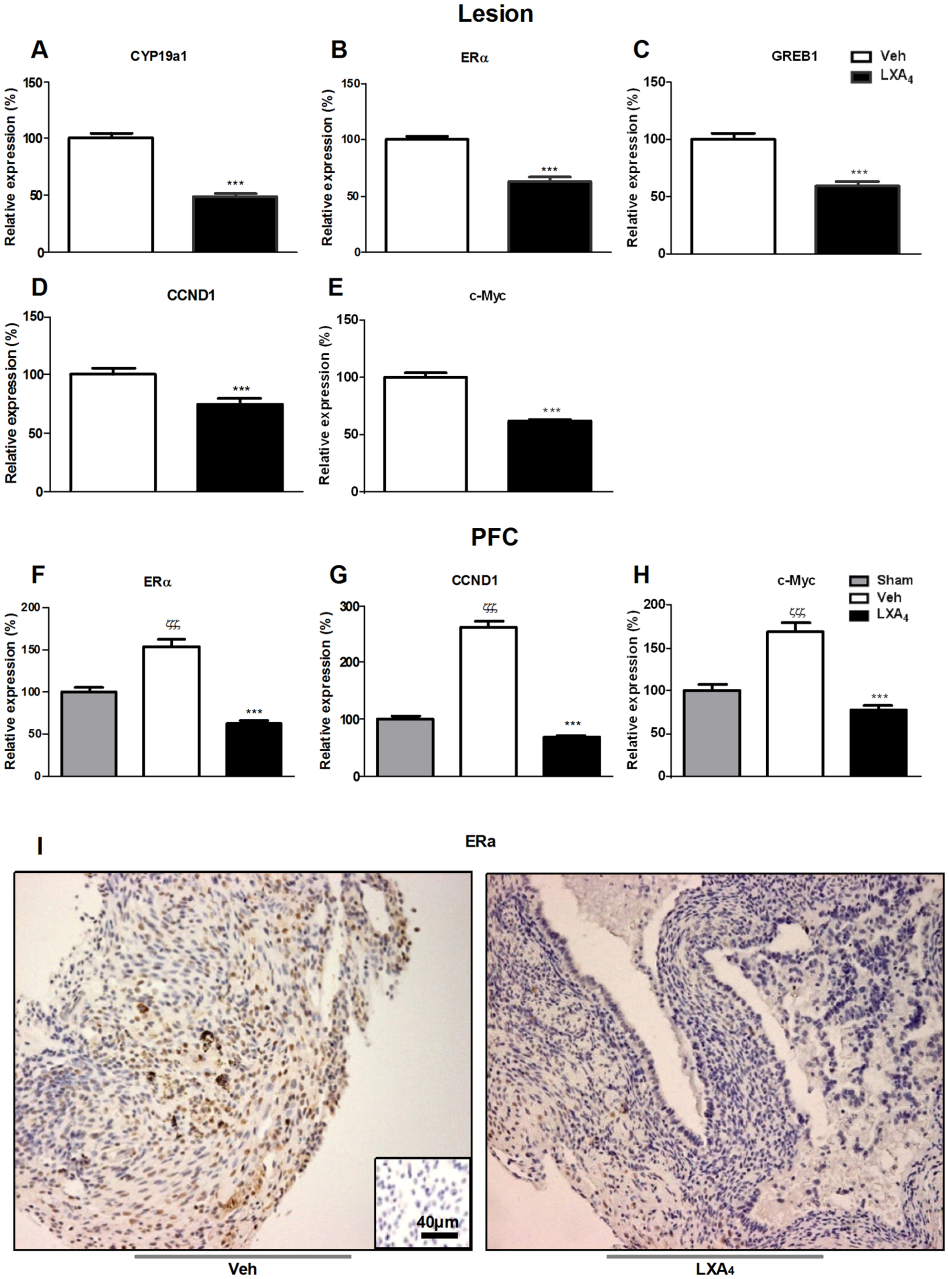
Mediators implicated in estrogen production and cellular proliferation in endometriotic lesions and PFCs are inhibited by LXA_4_ treatment. mRNA was extracted from endometriotic lesions and PFCs and subjected to qPCR analysis to assess transcript levels of **A.** CYP19a1, **B.** ERα, **C.** GREB1, **D.** CCND1, **E.** c-Myc and **F.** ERα, **G.** CCND1, **H.** c-Myc, respectively. Results were normalized to GAPDH. Ten mice were used per group (Sham, Veh-, LXA_4_-treatment) and sacrificed 21 days after surgical induction of endometriosis. Data are presented as mean ± SEM. (***p<0.001 compared to Veh; ζζζ p<0.001 compared to Sham). **I.** Immunohistochemical staining for ERα was performed on transverse sections of endometriotic lesions from Veh- (left panel) and LXA_4_-treated (right panel) mice. The negative control is shown in the inset.

Estrogen production occurs from C19 steroids by the enzymatic activity of aromatase (CYP19a1) and is involved in the establishment and maintenance of endometriotic lesions [2], [33]. Interestingly, LXA_4_ downregulated CYP19a1 (Figure 6A) in endometriotic lesions. Aromatase mRNA expression was undetectable in PFCs and it was also not possible to detect estrogens in the corresponding peritoneal fluid using a sensitive LC MS/MS-based method (data not shown). Lesions do not provide sufficient material for such analyses. Estrogen receptor alpha (ERα) expression was likewise attenuated by LXA_4_ in lesions and PFCs (Figure 6B, 6F). Immunohistochemical analyses of ERα in lesions yielded similar results at the protein level (Figure 6I). Furthermore, LXA_4_ augmented PR expression in lesions (p<0.001, data not shown), while PR was undetectable in PFCs. Of note, as assessed by performing vaginal smears, cycling was not impacted in LXA_4_-treated mice (data not shown).

Cellular proliferation is a key factor underlying endometriosis development and progression [34]. Estrogens could promote endometrial tissue proliferation by activating specific genes involved in the cell cycle. An important gene in this respect is cyclin D1 (CCND1), involved in the regulation of G/S transition during the cell cycle [35]. c-Myc, a transcription factor, regulates a wide array of genes implicated in cell growth, proliferation and loss of differentiation [36], [37]. Notably, LXA_4_ attenuated mRNA expression of both CCND1 (Figure 6D and 6G) and c-Myc (Figure 6E and 6H) in endometriotic lesions and PFCs, respectively. In addition, the expression of the gene regulated by estrogen in breast cancer 1 (GREB1), involved in cell proliferation [38], was reduced in endometriotic lesions in LXA_4_-treated mice (Figure 6C). As would be expected for an epithelial molecule [38]–[40], GREB1 mRNA was undetectable in PFCs. These data provide further insight into the mechanisms leading to decreased lesion size that we observed upon LXA_4_ treatment.

### LXA_4_ treatment also results in reduced progression of established endometriotic lesions

**Figure 7:**
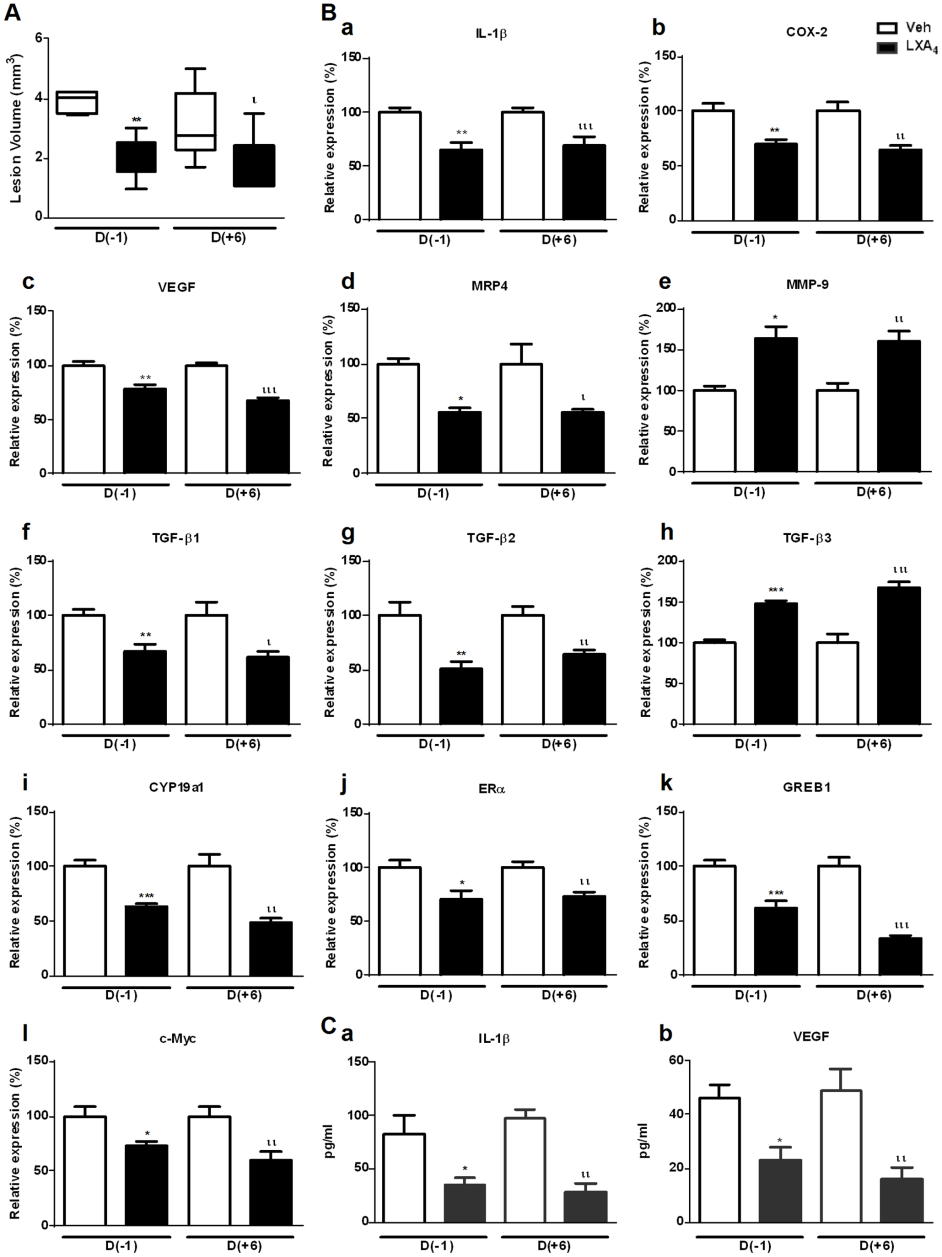
LXA_4_ reduces the progression of established endometriosis by similar mechanisms. Peritoneal endometriosis was surgically induced in mice divided into 3 groups (n = 7/group): Veh or LXA_4_ (5 μg/kg/mouse once daily) was administered from the day prior to surgery D(−1), or started at postoperative day 6 D(+6). Treatment was continued until day 21 after surgery, when the mice were sacrificed. **A.** Endometriotic lesion volume expressed as mean mm^3^ ± SEM (**p<0.01). **B. a.** IL-1β, **b.** COX-2, **c.** VEGF, **d.** MRP4, **e.** MMP-9, **f.** TGF-β1, **g.** TGF-β2, **h.** TGF-β3, **i.** CYP19a1, **j.** ERα, **k.** GREB1 and **l.** c-Myc mRNA expression was quantified in endometriotic lesions by qPCR and normalized to GAPDH. **C. a.** IL-1β and **b.** VEGF protein concentrations in the peritoneal fluid were measured by ELISA and expressed as pg/ml. All data are presented as mean ± SEM. Unpaired *t*-tests were performed for comparisons with the corresponding Veh-treated group (*p<0.05 **p<0.01, ***p<0.001, ^ι^p<0.05, ^ι ι^p<0.01, ^ι ι ι^p<0.001).

Patients attending Gynecology clinics because of pain and/or fertility problems already have some degree of endometriosis. To reflect this scenario and to determine whether our findings were therapeutically relevant, we investigated the effect of once-daily LXA_4_ treatment on established lesions. LXA_4_ treatment was thus commenced either the day prior to surgery (D−1) or on postoperative day 6 (D+6), a time-point when lesions on the peritoneal wall are already well developed. Interestingly, LXA_4_ reduced endometriotic lesion volume in established disease, i.e. when given from D+6 (Figure 7A). We then selected representative genes for the different pathways that we had observed to be implicated in the pathogenesis of endometriosis in our *in vivo* model, namely inflammation, angiogenesis, PGE_2_ production and transport, matrix remodeling and estrogen signaling. As observed when LXA_4_ was administered on D−1, IL-1β, COX-2, VEGF, MRP4, TGF-β1, TGF-β2, CYP19a1, ERα, GREB1 and c-Myc expression were significantly attenuated whereas MMP-9 and TGF-β3 were upregulated in endometriotic lesions in established endometriosis (Figure 7Ba-l). Gene expression profiling of PFCs yielded similar results (data not shown). IL-1β and VEGF protein levels in the peritoneal fluid were also significantly reduced by LXA_4_ treatment in both *de novo* and established disease (Fig. 7Ca, b).

## Discussion

In the present study we examined the effect of LXA_4_ on the IL-1β-COX-2-PGE_2_-VEGF axis as well as on ERα and E2-regulated genes implicated in an experimental endometriosis model. It has been reported that the proliferation of ectopic endometrial tissue is influenced by various cytokines, lipid mediators and growth factors present in the peritoneal fluid. The ectopic endometrium itself may also produce pleiotropic factors promoting its survival and growth [2], [7]. The mechanisms by which endometrial tissues attach, proliferate, avoid immune surveillance and derive a local vasculature at ectopic sites are not well understood. We thus investigated various mediators in the peritoneal fluid, as well as in PFCs and endometriotic lesions in a mouse model of disease.

Lipoxins are endogenous eicosanoids produced during inflammatory events by platelet-leukocyte and leukocyte-epithelial interactions. Their anti-inflammatory and pro-resolving properties have been well described [41]. Using a surgically induced endometriosis model [25], [42], we first showed that treatment with LXA_4_ reduced the volume of endometriotic lesions in part by attenuating pro-inflammatory (IL-1β and IL-6) and pro-angiogenic (VEGF) mediators in endometriotic lesions and PFCs. Our results confirm and extend previous reports describing the inhibitory effect of LXA_4_ and a stable analogue on the development of endometriotic lesions [22], [23], [43], using a different murine experimental model where endometrial tissue fragments were injected into the peritoneum.

Inflammatory, hypoxic and metabolic conditions lead to HIF activation and the induction of VEGF expression [44], a feature of endometriosis pathology [45], [46]. We observed this pathway to be activated in endometriosis and LXA_4_ attenuated the expression of HIF-1α mRNA in endometriotic lesions and PFCs. Among the angiogenic factors, VEGF-A is the most studied and patients with endometriosis have increased VEGF levels in their peritoneal fluid compared to the peritoneal fluid and eutopic endometrium of healthy women [46], [47]. VEGF is thus involved in the pathogenesis of endometriosis and the inhibition of VEGF and/or its receptor may be a potential therapeutic target for the treatment of endometriosis as investigated in various animal models [45], [48]–[50]. In line with these findings, we observed that LXA_4_ significantly downregulated VEGF-A mRNA levels in endometriotic lesions and PFCs, as well as VEGF-A protein in the peritoneal fluid. These data suggest that LXA_4_'s anti-angiogenic capacity results in decreased lesion size, corroborating previously published data [22], [23].

COX-2, the rate limiting enzyme in PGE_2_ biosynthesis, is increased in ectopic and eutopic endometrial tissues of endometriosis patients compared to healthy controls by cytokines such as IL-1β [51], [52]. PGE_2_ in turn strongly induces CYP19a1 (Aromatase), thus creating a positive feedback mechanism that maintains elevated local estradiol levels [2], [53], [54]. We observed elevated COX-2 expression in the lesions and PFCs of Veh-treated compared to sham mice. COX-2 expression in PFCs and endometriotic lesions was down-regulated upon LXA_4_ treatment. This was confirmed by immunohistochemical analyses, revealing markedly more intense COX-2 staining in endometriotic stromal cells and macrophages within lesions of Veh-treated compared to LXA_4_-treated mice. Furthermore, as compared to Veh-treated mice, LXA_4_ decreased CYP19a1 expression in endometriotic lesions, this coincided with decreased expression of E2-regulated molecules involved in cellular proliferation. We have recently demonstrated that the PGE_2_ transporter MRP4, a potential biomarker, is upregulated in peritoneal endometriosis in women, and that its expression is attenuated by LXA_4_ *in vitro*, signaling via ERα [55]. Here, we confirm those findings *in vivo*. The expression of MRP4, which functions to actively transport PGE_2_ and other molecules into the extracellular environment [29], was reduced in endometriotic lesions and PFCs, consistent with the decreased PGE_2_ peritoneal fluid levels. These findings support the hypothesis that LXA_4_ controls endometriotic lesion growth by inhibiting PGE_2_ production and it's efflux into the extracellular space where it could bind its receptors and initiate signaling pathways, resulting, in turn, in blunted estrogen production and proliferative mediator expression. It is tempting to speculate that the attenuated CYP19a1 expression we observed is due to decreased local PGE_2_ levels, this novel observation warrants further study to determine whether LXA_4_ downregulates CYP19a1 expression via this mechanism or via direct transcriptional repression.

Estrogen production and signaling are considered central to the pathogenesis of endometriosis [2], [7], [25]. Accordingly, in our surgically induced model using mice with intact uteri and ovaries, we observed increased CYP19a1, ERα and COX-2 expression in the endometriotic lesions of Veh-treated mice as well as increased PGE_2_ peritoneal fluid levels compared to sham mice, which were reduced in LXA_4_-treated animals. As ERα is dynamically regulated, we studied estrogen signaling by assessing the impact of LXA_4_ on the downstream effectors c-Myc, CCND1 and GREB1, key regulators of cell cycle progression and cellular proliferation, whose dysregulated expression and activation has been implicated in the pathogenesis of human cancers [36], [37], [40], [56], and in endometriotic cell proliferation [57]–[59]. GREB1, which we have shown is upregulated in peritoneal endometriosis [39], was also identified in genome wide association studies as containg a risk locus [60].

c-Myc is additionally considered a key player in alternative macrophage polarization [61]. We and others have reported that these molecules were overexpressed in peritoneal lesions and eutopic endometrium of endometriosis patients in comparison to control subjects [39],[62],[63]. In PFCs, CCND1 and c-Myc expression was higher in Veh-treated mice compared to control sham mice, suggesting a role in disease development. Interestingly, LXA_4_ treatment attenuated the expression of c-Myc, CCND1 and GREB1 in endometriotic lesions and that of c-Myc and CCND1 in PFCs. ERα was also reduced in both compartments. This data is in line with with our recent characterization of LXA_4_ as an estrogen receptor ligand, which can inhibit endogenous E2-mediated signaling under certain circumstances in a manner similar to the weak estrogen Estriol [64], [65], with which it shares structural similarity [18]. Hence, these results indicate that LXA_4_ exerts a local anti-proliferative effect, possibly via ERα, a receptor essential for endometriosis development in mice [66]. ALX/FPR2 to which LXA_4_ binds with high affinity and whose expression is upregulated under inflammatory conditions in *in vitro* experimental models [67] as well as in tissue biopsies from patients with inflammatory diseases [68], was also increased in lesions after surgical induction of endometriosis as assessed by a previous gene array analysis [69]. This receptor was significantly attenuated by LXA_4_ treatment in both lesions and peritoneal fluid cells, indicating either a reduction in receptor expression on cells *in situ* or decreased migration of ALX/FPR2-positive cells into these compartments.

To further understand the progression of the disease and the beneficial effects of LXA_4_ treatment, we analysed the expression of TGF-β, a mediator involved in cell proliferation and differentiation as well as in tissue repair. TGF-β is highly expressed in the endometrium and is secreted by endometrial glands and macrophages [32], [70]. Patients with endometriosis have higher levels of TGF-β1 in the peritoneal fluid compared to disease-free subjects [10], [71] and higher TGF-β2 mRNA expression was reported in the ectopic endometriotic lesions [72]. In our study, LXA_4_ differentially regulated TGF-β isoforms. Upon LXA_4_ treatment, the expression of TGF-β1 and 2 was attenuated in endometriotic lesions and PFCs, whereas TGF-β3 expression was upregulated in the same compartments. These findings merit further investigation as, besides being a growth factor, TGF-β was shown to be essential in wound healing and tissue repair by inducing extracellular matrix components [73]. We also assessed the activity of MMPs, involved in extracellular matrix degradation and thereby likely contributing to the local invasiveness of the lesions. Of note, Wu et al., reported that high levels of PGE_2_ in the peritoneal cavity of endometriotic patients inhibited MMP-9 activity, thus interfering with the clearance of endometrial tissue [21]. We observed elevated MMP-9 expression in endometriotic lesions, but not in PFCs, of LXA_4_-treated mice, where it was decreased. On a functional level, MMP-9 activity in this environment was increased, possibly contributing to decreased lesion size. However, these molecules are regulated in a complex manner and our findings likely reflect the expression patterns of the various TGF-β isotypes and MMP subtypes which may be both differentially regulated and compartmentalized as a function of specific anatomical sites and cell types during the course of disease.

Current standard medical treatments for endometriosis include therapies that control the production of estrogens alone or in combination with anti-inflammatory drugs [74], [75]. However, as well as being partially effective these treatments have considerable systemic side-effects including a hypo-estrogenic state that may lead to osteoporosis, as well as impaired fertility. Much published data suggest that endometriosis is a local rather than a systemic phenomenon with the induction of a local microenvironment in the peritoneal cavity which promotes ectopic lesion growth. We have demonstrated that LXA_4_ inhibits the progression of endometriosis via multiple local mechanisms. In this context, LXA_4_ regulates mediators which are responsible for diverse biological activities such as inflammation (IL-1β, IL-6, COX-2, PGE_2_), cell survival and proliferation (PGE_2_, CYP19a1, CCND1, c-Myc, GREB1), cell adhesion and invasion (MMPs, TGF-β) and angiogenesis (HIF-1α, VEGF, TGF-β). Further investigations are necessary to delineate whether the anti-inflammatory effects of LXA_4_ are directly exerted on epithelial and stromal cells and/or via trafficking immune cells. As macrophages are major sources of pro-inflammatory cytokines and PGE_2_, the subsets in the peritoneal cavity and in the lesions necessitate elucidation.

LXA_4_ inhibited the progression of endometriosis by attenuating local estrogen-mediated signaling in endometriotic lesions and in peritoneal fluid cells, and most likely reducing estrogen production. This, without affecting the estrous cycle as was also previously reported [22] or evoking any other noticeable adverse effects. We also made the novel and clinically relevant observation that LXA_4_ was effective when administered in the context of established disease. Taken together, LXA_4_ and its stable analogues warrant further consideration as potential therapeutics for the treatment of peritoneal endometriosis.
